# A rare human variant that disrupts GPR10 signalling causes weight gain in mice

**DOI:** 10.1038/s41467-023-36966-3

**Published:** 2023-03-15

**Authors:** Fleur Talbot, Claire H. Feetham, Jacek Mokrosiński, Katherine Lawler, Julia M. Keogh, Elana Henning, Edson Mendes de Oliveira, Vikram Ayinampudi, Sadia Saeed, Amélie Bonnefond, Mohammed Arslan, Giles S. H. Yeo, Philippe Froguel, David A. Bechtold, Antony Adamson, Neil Humphreys, Inês Barroso, Simon M. Luckman, I. Sadaf Farooqi

**Affiliations:** 1 University of Cambridge Metabolic Research Laboratories and NIHR Cambridge Biomedical Research Centre, Wellcome-MRC Institute of Metabolic Science, Addenbrooke’s Hospital, Cambridge, CB2 0QQ UK; 2 Division of Diabetes, Endocrinology and Gastroenterology, Faculty of Biology, Medicine and Health, University of Manchester, Manchester, UK; 3 Department of Metabolism, Digestion and Reproduction, Imperial College London, London, UK; 4 Inserm UMR1283, CNRS UMR8199, European Genomic Institute for Diabetes (EGID), Institut Pasteur de Lille, Lille University Hospital, Lille, 59000 France; 5 Université de Lille, Lille, 59000 France; 6 School of Life Sciences, Forman Christian College, Lahore, Pakistan; 7 MRC Metabolic Diseases Unit, Wellcome-MRC Institute of Metabolic Science, Addenbrooke’s Hospital, Cambridge, CB2 0QQ UK; 8 Genome Editing Unit, Faculty of Biology, Medicine and Health, University of Manchester, Manchester, UK; 9 MRC Epidemiology Unit, University of Cambridge, Cambridge, UK; 10 Exeter Centre of Excellence for Diabetes Research (ExCEED), University of Exeter Medical School, Exeter, UK

**Keywords:** Clinical genetics, Endocrine system and metabolic diseases

## Abstract

Disruption of brain-expressed G protein-coupled receptor-10 (GPR10) causes obesity in animals. Here, we identify multiple rare variants in *GPR10* in people with severe obesity and in normal weight controls. These variants impair ligand binding and G protein-dependent signalling in cells. Transgenic mice harbouring a loss of function *GPR10* variant found in an individual with obesity, gain excessive weight due to decreased energy expenditure rather than increased food intake. This evidence supports a role for GPR10 in human energy homeostasis. Therapeutic targeting of GPR10 may represent an effective weight-loss strategy.

## Abstract (web summary)

The brain-expressed receptor GPR10 is involved in energy homeostasis in mice. Here the authors identify rare loss of function variants in GPR10 in people with severe obesity and showed that one of these variants causes obesity when modelled in mice, suggesting that future studies could explore GPR10 as a potential target for weight-loss therapy.

## Introduction

There is a substantial unmet need for new weight-loss treatments to reduce the morbidity and mortality associated with obesity^1^. Human genetic studies have established that some of the molecules known to regulate energy homeostasis in rodents contribute to human physiology, validating these molecules and pathways as therapeutic targets^2^.

G protein-coupled receptor 10 (GPR10) is a centrally expressed G protein-coupled receptor (GPCR) which acts as the cognate receptor for prolactin-releasing peptide (PrRP), an evolutionarily conserved RF-amide peptide^3–5^. PrRP reduces food intake and increases energy expenditure when administered centrally in rodents. Moreover, PrRP-containing neurons in the dorsomedial nucleus of the hypothalamus (DMH) have been shown to play a key role in mediating the thermogenic effects of the adipocyte-derived hormone leptin^6,7^. There has been interest in targeting GPR10 for weight-loss therapy. Indeed, systemically administered palmitoylated PrRP analogues reduce body weight in mouse models of diet-induced obesity, potentially through central mechanisms^8,9^.

In this study, we identified multiple rare variants in *PRLHR* (Prolactin Releasing Hormone Receptor), also known as *GPR10*, in cases with severe obesity and ancestry-matched controls, which we showed in cellular studies, impaired ligand binding and G protein-dependent signalling. To test the physiological consequence of human variants on body weight, we generated mice harbouring a functional *GPR10* variant found in an individual with severe obesity. We found that transgenic mice gained excessive weight due to decreased energy expenditure rather than increased food intake. Cumulatively, these studies indicate that therapeutic targeting of GPR10 may be an effective weight-loss strategy.

## Results

### Characterisation of rare variants in *GPR10*

**Fig. 1:**
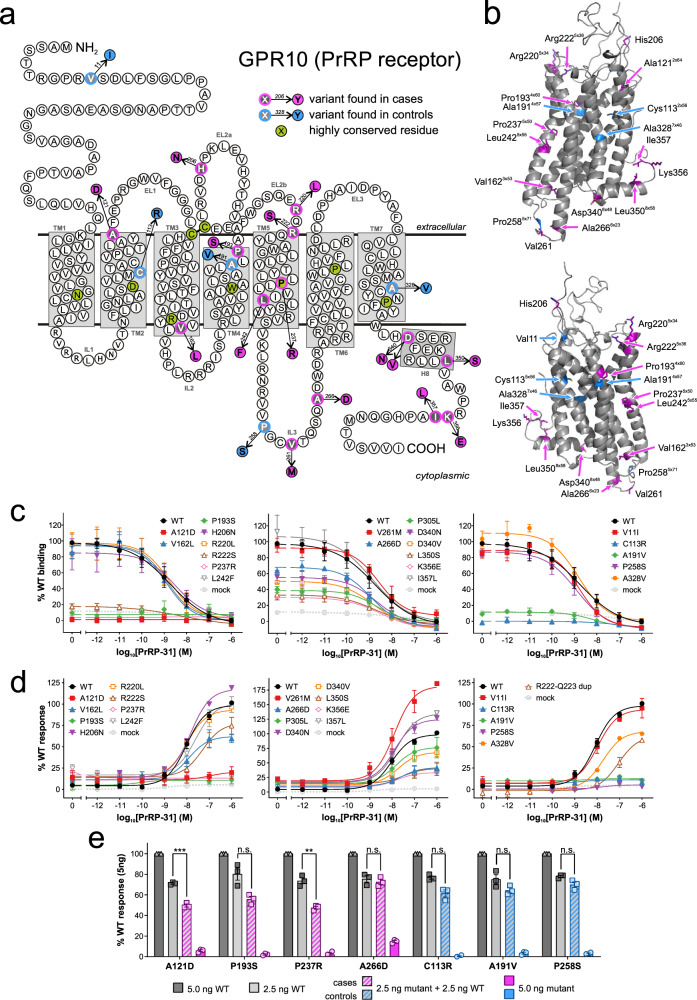
Functional characterisation of rare variants in *GPR10* identified in cases with severe obesity and controls. **a** Rare variants identified in individuals with severe early onset obesity (magenta) and in controls (blue) shown on a schematic of the GPR10 protein and **b** on a structural model. For residues located in α-helices, a generic residue number according to a structure-based GPCRdb numbering scheme shown in subscript^18^. Wild-type (WT) and mutant forms of GPR10 were studied in cells alongside a low frequency variant (P305L); mock transfected cells served as a negative control. Effects on ligand (PrRP-31)-induced binding **c** and **d** receptor-mediated activation of Gα_q/11_-regulated inositol triphosphate signalling in transiently transfected COS-7 cells. Data shown as sum curves from 3-9 experiments normalized to WT maximal binding/response±SEM. **e** COS-7 cells were transiently co-transfected with WT and varying amounts of mutant GPR10 to investigate dominant negative effects on PrRP-31-induced Gα_q/11_-regulated inositol triphosphate signalling. Mean±SEM of 4 experiments are shown; differences between means for 2.5 ng WT alone and with additional 2.5 ng mutant GPR10 were compared with Students t-test, ns – not significant; ^**^p < 0.01; ^***^p < 0.001 (based on data shown in Supplementary Fig. 1, panels d-i). Source data are provided as a Source Data file.

To investigate the potential contribution of genetic variation in *GPR10* to severe human obesity, we interrogated exome sequencing and targeted resequencing data on 2548 European ancestry individuals with severe, early-onset obesity recruited to the Genetics of Obesity study (GOOS; www.goos.org.uk) (mean Body Mass Index (BMI) standard deviation score>3; age of onset <10 years); mutations in known obesity genes had been excluded. We also studied 1117 ancestry-matched controls analysed using the same methods^10^. We identified 15 rare heterozygous variants in *GPR10* in 17 unrelated individuals with obesity (A266D was found in three unrelated individuals); we also found five rare variants in controls (Odds Ratio [95% confidence intervals] =1.5 [0.5–5.2], *p* = 0.5, Fisher’s exact test) (Fig. 1a, b; Supplementary Table 1). As a limited number of family members were available for study, the mode of inheritance is unclear (six probands inherited the *GPR10* variant from a parent with overweight/obesity; one proband inherited the variant from a parent who was normal weight. Variant carriers had a number of other clinical features including anxiety, impaired memory and impaired pain sensation (Table 1).

**Table 1:** Phenotypes seen in carriers of rare variants in GPR10

Variant	Age (years)	Weight (kg)	Height (cm) [SDS]	BMI (kg/m^2^) [SDS]	Temp (^o^ C)	Heart rate (bpm)	BP (mmHg)	Glucose (mmol/l)	Insulin (pmol/l)	Neurobehavioural conditions
A121D*	13.7	107.0	167.5 [0.9]	38.1 [3.6]	N/A	N/A	N/A	N/A	N/A	
V162L*	14.9	78.9	153.4 [−1.8]	33.5 [3.1]	N/A	N/A	N/A	4.6	100	
P193S*	18.4	109.0	169.9 [1.0]	37.7 [3.3]	36.4	54	111/56	4.2	116	Night terrors/sleep walks, anxiety, depression, self-harm, impaired memory
P193S	52.9	88.7	180.7	27.2	36.2	71	127/74	4.6	23	Anxiety, depression, impaired memory, impaired pain sensation
H206N*	4.7	55.2	121.0 [3.0]	37.7 [6.5]	N/A	N/A	85/55	4.8	N/A	
R220L*	15.7	129.2	155.6 [−1.2]	53.4 [4.4]	36.4	85	103/55	14.2#	69	Aggression, low mood
R220L	54.2	91.8	173.7	30.4	36.0	66	107/61	8.4#	50	Anxiety, low mood, emotional lability, impaired memory
R220L	22.5	80.0	160.2	31.2	36.1	75	99/55	4.7		
R222S*	24.7	91.4	166.7	32.9	36.8	45	124/67	4.7#	38	Anxiety, depression, autism-like
R222S*	16.2	119.6	161.9 [−0.2]	45.6 [4.0]	N/A	N/A	N/A	4.1	293	
L242F*	10.1	78.5	144.1 [0.8]	37.8 [3.9]	N/A	N/A	N/A	4.1	N/A	
A266D*	26.0	116.4	161.1	44.9	36.9	58	113/64	4.5	99	Impaired memory
A266D	8.6	28.0	124.5 [−1.1]	18.1 [0.9]	36.7	73	100/59	4.2	45	Hyperactive, impaired memory, impaired pain sensation
A266D*	8.7	68.0	132.3 [0.2]	38.9 [4.3]	37.0	81	134/78	4.3	103	Aggression, fearless, emotional lability
A266D	9.8	64.4	144.6 [1.2]	30.8 [3.3]	36.6	79	125/69	4.3	120	
A266D*	11.4	102.3	145.3 [−0.2]	48.5 [4.3]	N/A	N/A	N/A	4.7	225	
D340N*	12.4	95.7	161.2 [1.3]	36.8 [3.5]	N/A	N/A	N/A	5.1	193	
L350S*	17.7	105.8	170.3 [1.1]	36.5 [3.2]	36.8	72	119/57	4	133	Impaired memory
L350S	48.4	71.0	170.7	24.4	36.5	51	130/68	3.6	7	

Data for individuals with severe obesity with *GPR10* variants where this was available. *BMI* Body Mass Index; age and gender-adjusted height and BMI Standard Deviation Scores (SDS) included for children up to age 18 years; fasting plasma insulin (0–60 pmol/l); ^*^ denotes proband, ^#^denotes type 2 diabetes and/or treated with metformin, ^o^C temperature (temp) in degrees Celsius, heart rate in beats per minute (bpm) and blood pressure (BP) in millimetres of mercury (mmHg) were measured in the rested fasted state, N/A indicates data not available.

To test whether the rare variants in *GPR10* might have functional consequences, we studied all 20 variants identified in cases with obesity and/or controls in cells transiently transfected with constructs encoding wild-type (WT) or mutant human GPR10 (Fig. 1a–e; Supplementary Table 2). We also studied a previously reported low frequency variant in *GPR10*, P305L (Minor Allele Frequency, MAF = 5%). Whilst this variant has not been associated with BMI, it has been associated with reduced systolic and diastolic blood pressure in a previous population based study^11^. None of the GPR10 mutants reduced cell surface expression measured by enzyme-linked immunosorbent assay (ELISA) (Supplementary Fig. 1a). In competitive radioligand binding assays, [^125^I]-labelled PrRP-31 did not bind to 6 of 20 GPR10 mutants. For some of these mutants, cell surface expression was not affected. As a number lie in the extracellular domain of the receptor, these amino acid substitutions may impair the conformation of the ligand binding pocket. For a further 7 of 20 mutants, the maximal amount of radioligand bound was significantly reduced (Fig. 1c), but none of the mutants affected binding affinity (IC_50_; Supplementary Table 2). This discrepancy may be partially explained by experimental methods (amplified signal detection in cell surface ELISA vs ratiometric measurements of receptor density in competition radioligand binding assays). Saturation binding studies may provide further insights into the mechanisms underlying these observations.

We next measured canonical Gα_q/11_-coupled signalling by quantifying ligand-induced inositol triphosphate turnover. Ten of 20 GPR10 mutants (7 of 15 in cases; 3 of 5 in controls) caused a loss of function (LoF) in this assay (Fig. 1d). In HEK293 cells stably expressing WT GPR10, we showed that GPR10 activation inhibits basal and forskolin-induced cyclic adenosine monophosphate (cAMP) accumulation (Supplementary Fig. 1b) consistent with Gα_i/o_–coupled signalling. We found that twelve of twenty GPR10 mutants (8 of 15 in cases, 4 of 5 in controls) impaired Gα_i/o_–coupled signalling measured using an inositol triphosphate turnover assay in the presence of Gα_Δ6qi4myr_ chimeric G-protein^12^ (Supplementary Fig. 1c). In these assays, the proportion of variants that exhibit LoF did not differ significantly between cases and controls (*p* > 0.05).

LoF missense *GPR10* variants found in heterozygous form may impact the phenotype through haplo-insufficiency or by having a dominant negative effect, where the mutated receptor inhibits signalling by the WT receptor. In preliminary studies, we studied seven mutants that significantly impaired Gα_q/11_-coupled signalling, in COS-7 cells co-transfected with varying doses of WT and mutant GPR10. Two of the four complete LoF variants found in cases (A121D, P237R), but none of the three LoF variants found in controls (Fig. 1e and Supplementary Fig. 1d–i) exhibited dose-dependent inhibition of signalling by WT GPR10. Further studies will be needed to examine the potential structural and molecular mechanisms which may underlie these effects. Cumulatively, we found that the majority of *GPR10* variants found in cases with severe obesity (11 of 15) and in controls (4 of 5), caused LoF in one or more cellular assay.

In a further study, we interrogated 581 exomes from unrelated probands with severe obesity from consanguineous families of Pakistani origin. In this cohort we identified a homozygous variant in the gene encoding GPR10 (NM_004248.3: c.665_670dup / p.R222_Q223dup) in a 5-year old girl with hyperphagia and weight gain from the age of 2.5 years, resulting in severe obesity (BMI SDS: 5.5). On a follow-up examination at the age of 8.5 years, the proband was reported to suffer from extended periods of anxiety and depression lasting for as many as 10 days (there was no history of developmental delay). Both parents were heterozygous for the variant which is found at MAF of 0.003512 in South Asian exomes (gnomAD v2.1.1). We characterised the function of this mutation in cells; the variant resulted in a partial loss of function (Fig. 1d).

### *Gpr10*^-/-^ knockout mice show increased weight gain on chow diet

**Fig. 2:**
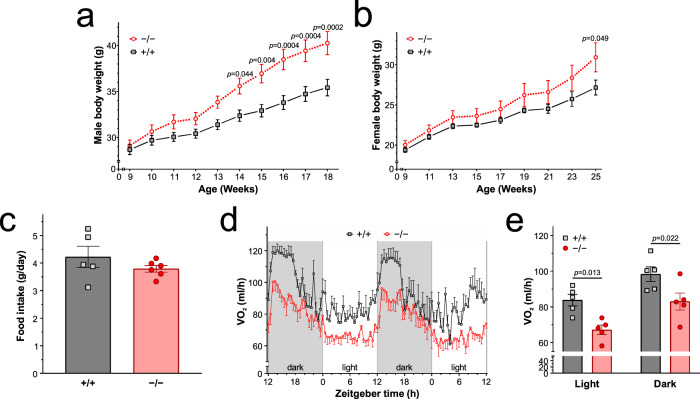
Obesity in *Gpr10*^-/-^ knockout mice. **a, b** Body weight curves for male and female homozygous wild-type *Gpr10*^+/+^ (grey squares) and *Gpr10*^-/-^ null mice (red circles) (males, *n* = 21-25 per group; females, *n* = 6 per group). Data are presented as mean±SEM and analysed with a mixed-effects analysis followed by Sidak’s multiple-comparison *post hoc* test. **c** Daily food intake (g) in 6 week old homozygous wild-type *Gpr10*^+/+^ (grey squares; *n* = 5) and *Gpr10*^-/-^ null mice (red circles; *n* = 6) fed on normal chow. Data are presented as mean±SEM and comparisons made using two-tailed Student’s *t*-test. **d, e** Oxygen consumption (VO_2_, ml/h) for male wild-type *Gpr10*^+/+^ (grey squares) and *Gpr10*^-/-^ null mice (red circles) at 6 weeks across the light-dark cycle (*n* = 5 in each group). Data are presented as mean±SEM and comparisons made using two-way ANOVA followed by Bonferroni’s multiple-comparison *post hoc* tests. Source data are provided as a Source data file.

Given their rarity, it is difficult to establish whether LoF human variants affecting GPR10 can cause obesity. We therefore set out to test directly whether a rare human variant in *GPR10* found in an individual with obesity could cause weight gain in mice. We first generated *Gpr10*^-/-^ null mice with a 54-base pair targeted deletion within the coding region and found that on a chow diet, both male and female mice exhibited increased weight gain compared with littermate controls (Fig. 2a, b). Male mice were significantly obese at 14-weeks age; female mice at 25-weeks of age. Importantly, whereas no difference in food intake was seen (Fig. 2c), pre-obese male *Gpr10*^*-/-*^ null mice exhibited reduced energy expenditure, measured as a difference in oxygen consumption (VO_2_) by indirect calorimetry (Fig. 2d, e); this effect persisted in older *Gpr10*^*-/-*^ null mice (Supplementary Fig. 2) compared with wild-type controls. These findings are in keeping with previous reports of obesity in *Gpr10*^-/-^ mice and the OLETF strain of rats, which carry a mutation in *Gpr10*^13–15^.

### Knock-in *Gpr10*^P193S^ mice develop obesity

**Fig. 3:**
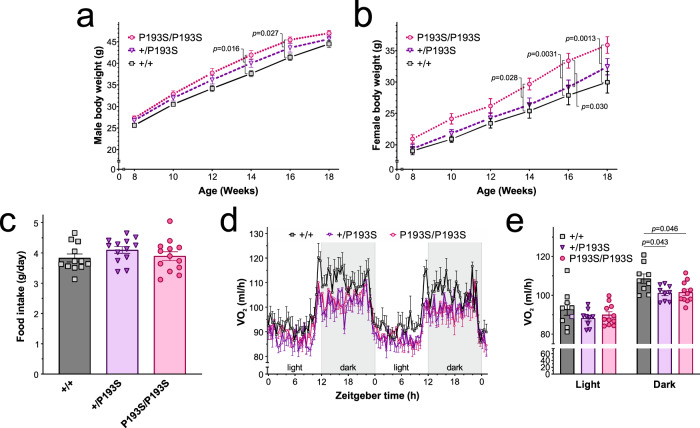
Obesity in *Gpr10*^P193S/P193S^ mutant mice. **a, b** Body weight curves of wild-type (^+/+^; grey squares), heterozygous (^+/P193S^; purple triangles) and homozygous *Gpr10*^P193S/P193S^ (pink circles) male (*n* = 9, 18 and 7 respectively) and female mice (*n* = 8, 8 and 5 respectively) maintained on 60% high energy diet from 8 weeks. Data are presented as mean± SEM and were analysed with a mixed-effects analysis followed by Sidak’s multiple-comparison *post hoc* test. **c** Daily food intake (g) in wild-type (^+/+^; grey squares), heterozygous (^+/P193S^; purple triangles) and homozygous *Gpr10*^P193S/P193S^ (pink circles) male mice aged 8 weeks on normal chow (*n* = 12, 13 and 13 respectively). **d, e** Oxygen consumption (VO_2_, ml/hr) across the light-dark cycle in male wild-type (^+/+^; grey squares), heterozygous (^+/P193S^; purple triangles) and homozygous *Gpr10*^P193S/P193S^ (pink circles) mice on standard chow at 8 weeks (*n* = 9, 9 and 11 respectively). Data are presented as mean ± SEM and comparisons made using two-way ANOVA followed by Bonferroni’s multiple-comparison *post hoc* tests. Source data are provided as a Source data file.

We then generated a knock-in mouse model of the human *GPR10* variant (*Gpr10*^P193S^) (Fig. 3 and Supplementary Fig. 3). This mutation affects a residue that is highly conserved across species and among Family A GPCRs^16^, is located at the extracellular end of transmembrane domain 4 and is predicted to affect the ligand binding pocket. As detailed above, GPR10^P193S^ shows impaired PrRP-31 binding (Fig. 1c) and complete loss of receptor activation in cells (Fig. 1d and Supplementary Fig. 1c).

We found that *Gpr10*^P193S^ homozygous mutant mice were of a similar weight to wild-type littermates at the age of 8 weeks (Fig. 3a, b), before they were placed on a high-energy diet. Both male and female mutant mice exhibited greater weight gain than wild-type littermates (Fig. 3a, b), with a distinct gene dose effect between homozygous (*Gpr10*^P193S/P193S^), heterozygous (*Gpr10*^+/P193S^) and wild-type (*Gpr10*^+/+^) littermates. At 16 weeks, the difference in weight between *Gpr10*^P193S/P193S^ and wild-type littermates was comparable to that between *Gpr10*^*-/-*^ and *Gpr10*^*+/+*^ mice (~5 g). As with *Gpr10*^*-/-*^ mice, pre-obese male *Gpr10*^P193S^ mice did not exhibit a difference in daily food intake (Fig. 3c), but did exhibit lower energy expenditure when compared with weight-matched littermates (Fig. 3d, e). Cumulatively, these findings support the importance of GPR10 in the regulation of energy expenditure and weight regulation.

### Common variants at the *GPR10* locus

It is challenging to establish whether *GPR10* variants that do not display Mendelian inheritance are associated with severe obesity, as studies in tens of thousands of cases with severe obesity and controls would be needed^17^ and cohorts of this scale do not exist. To investigate whether common variants in the region of *GPR10 (PRLHR)* are associated with anthropometric or metabolic traits, we searched for GWAS and PheWAS associations and putative prioritisation of *GPR10* as a causal gene using the Open Targets Genetics platform (Supplementary Tables 3–6). We found that GPR10 is prioritised at loci associated with percentage body fat (lead variant rs4752183, *p* = 2  × 10^−8^; GPR10 locus-to-gene Score=0.7) and other anthropometric traits (Supplementary Table 3). The low frequency coding variant (P305L, rs8192524-A; allele frequency 5%) is nominally associated with higher percentage body fat and other anthropometric traits in single-variant analyses of UK Biobank genotypes (Supplementary Table 4) and exomes (Supplementary Table 5). Gene-based analyses combining low frequency and rare missense variants in *GPR10* tend towards an association with higher mean body fat percentage (*p* = 0.02, beta=0.001, Burden test; *p* = 0.007, SKAT-O; SAIGE-GENE mixed model, UK Biobank 280,000 Non-Finnish European exomes; https://genebass.org) and other anthropometric traits (Supplementary Fig. 4a, b; Supplementary Table 6).

An important related question is whether rare variants in *GPR10* observed in the population contribute to severe obesity (BMI > 40 kg/m^2^). To address this question, we interrogated rare variants (MAF < 0.1%) in *GPR10* in the subset of approximately 150,000 unrelated European ancestry exomes from UK Biobank (Methods). We observed a nominal association between rare coding variants in *GPR10* (MAF < 0.1%, 139 variants) and severe obesity (BMI > 40 kg/m^2^, *N* = 2725/152837 people) (*p* = 0.03, Robust SKAT-O; *p* = 0.04, Robust Burden test) with odds ratio >1 (*N* = 18/629 carriers, 2707/152208 non-carriers, OR (95% CI) = 1.63 (0.96,−2.6)). We did not observe an association with continuous BMI (*p* = 0.6, SKAT-O) nor with BMI dichotomised at the upper quartile (BMI > 29.8 kg/m^2^; *p* = 0.8, Robust SKAT-O). Five rare variants had nominal single-variant association with severe obesity (BMI > 40 kg/m^2^; *p* < 0.05,) all of which had odds ratio >1 and were located in transmembrane domain 4/extracellular loop 2a (TM4/EL2a) (V196G, R209S, A177P) or C-terminal domain (D340N, I357R; both are residues where variants were identified in our study of people with severe early-onset obesity) (Supplementary Fig. 4). Region-based case-control tests for severe obesity (BMI > 40 kg/m^2^) suggest an association with rare coding variants (MAF < 0.1%) in EL2a/TM4 (OR (95% CI) = 5.7 (2.0–13), *p* = 0.001, Fisher’s exact; *N* = 6/64 carriers, 2719/152773 non-carriers; *p* = 0.001, Robust SKAT-O) (Supplementary Table 7 and Supplementary Fig. 4d). The variant P193S, identified in the GOOS cohort and shown to cause obesity when studied in mice, lies at the EL2a boundary with transmembrane domain 4 (Supplementary Fig. 4e). These findings are intriguing and suggest that a subset of variants in the gene encoding GPR10 contribute to human weight regulation.

## Discussion

In conclusion, our data in humans and in mice, suggest that targeting GPR10 represents a potential therapeutic strategy for obesity. Further studies are needed to explore the potential consequences of targeting GPR10. An outstanding question relates to the interaction between GPR10 and the physiological response to stress. PrRP neurons in the medulla oblongata and/or in the dorsomedial hypothalamus are activated by a variety of stressful stimuli^5^. Central administration of PrRP activates corticotropin-releasing hormone neurons and oxytocin neurons in the hypothalamus and facilitates adrenocorticotropic hormone and oxytocin release into the systemic circulation^18,19^. Experiments in rodents suggest that PrRP has inhibitory effects on the neuroendocrine response to stress^20^. Here we show that human phenotypes associated with loss of function GPR10 mutations include anxiety, depression, impaired memory and impaired pain sensation (Table 1). While gaps in understanding remain, these observations suggest that GPR10 agonism may have beneficial effects on anxiety, depression and memory and may alter the perception of pain. As seen with other targets for weight loss therapy e.g. the Serotonin 2c receptor and the Cannabinoid 1 receptor^21,22^, the overlap between neural pathways that regulate weight and those that modulate other behaviours and cognitive phenotypes presents potential challenges for drug development. The transgenic mouse model of a human LoF GPR10 mutation generated here will enable further exploration of the role of GPR10 in coupling the stress response to changes in energy homeostasis.

## Methods

### Study design and approval

All genetic and clinical studies were approved by the Multi-Regional Ethics Committee and the Cambridge Local Research Ethics Committee (MREC 97/21 and REC number 03/103) and conducted in accordance with the principles of the Declaration of Helsinki. All participants, or their legal guardian for those aged under 16, provided written consent for all assessments; participants under the age of 16 provided oral assent. *GPR10* variant carriers were identified as part of genetic studies of individuals recruited to the Genetics of Obesity Study (GOOS), a cohort of 8000 individuals with severe early-onset obesity; age of obesity onset less than 10 years^19^. Severe obesity in GOOS was defined as a Body Mass Index (BMI, weight in kilograms divided by the square of the height in meters) standard deviation score ≥3 (standard deviation scores calculated according to the United Kingdom reference population). The details of the genetic analysis have been published previously^10^. Participants did not receive compensation for taking part in this study.

### Exomes from consanguineous families

We searched for rare *GPR10* homozygous variants in 581 exomes from unrelated probands (0.2–22 years of age) with severe, early onset obesity from consanguineous families of Pakistani origin. The details regarding the cohort and the genetic investigation have been published before^20^.

### UK Biobank 200 K exomes

This research has been conducted using the UK Biobank Resource under Application Number 53821. UKB analysis was based on interim UKB exomes releases, and future work will analyse a larger UKB exome release. We used the UK Biobank pVCF variant file (chromosome 10, block 34) from the OQFE exome pipeline (UK Biobank Field 23156; *N* = 200,629 exomes available to us (https://ukbiobank.dnanexus.com/)). Relatedness was obtained from the UK Biobank Genetic Data resource (ukbgene rel) and one person was excluded from each related pair among all the OQFE exomes (kinship ≥ 0.0442, KING, third-degree kinship or closer; subsetted the pairwise kinship results from ‘ukbgene rel’ to retain the pairs contained within OQFE exomes and excluded the individuals in column ‘ID2’; excluded *N* = 15,547 people). The resulting unrelated OQFE exomes were taken forward for analysis (*N* = 185,082). Exomes were further restricted to European genetic grouping (Field 22006) (*N* = 153,352).

We used Body mass index (BMI, kg/m^2^) obtained from the UK Biobank initial assessment visit (UK Biobank Field 21001, Instance 0) and this value was available to us for *N* = 184,294/185,082 unrelated exomes and 152,837/153,352 unrelated European exomes. The reported BMIs for selected individuals were checked for plausibility (ie. not obviously result of inaccurate derivation or recording of BMI) by inspecting other relevant measurements: height (Field 50), weight (Field 21002) and waist circumference (Field 48) at initial assessment, and related longitudinal measurements from repeat assessments where available.

Variant annotation was performed using Ensembl Variant Effect Predictor (VEP) (Ensembl release 102) and consequences defined with respect to Ensembl canonical transcript ENST00000239032. Variant filtering by minor allele frequency (MAF) was based on the maximum reported MAF among unrelated OQFE exomes, each gnomAD exome subpopulation (VEP field “gnomAD_exomes_POPMAX_AF”) and each 1000 Genomes subpopulation (VEP). We performed gene-based association tests for rare (MAF < 0.1%) non-synonymous exonic or splicing *GPR10* variants and severe obesity (BMI > 40 kg/m^2^, case:control ratio=1:53), for BMI dichotomised at the upper quartile of the unrelated exomes (BMI > 29.8 kg/m^2^, case: control ratio=1:3) and for continuous BMI.

Gene-based burden and SKAT-O tests were performed using the *SKATBinary_Robust* function for dichotomised BMI (or the *SKAT* function for continuous BMI) in R package SKAT v2.0.1 (method = ”Burden” or “SKATO” with default settings)^21^. The null models were calculated using *SKAT_Null_Model(y~X)* where the covariates matrix (*X*) contained age (Field 21003.0.0), sex (Field 31.0.0), ten genetic principal components (Fields 22009.0.1-10) and sequencing batch (UKB 50 K or 150 K exomes). Single-variant p-values are reported using the gene-based *SKATBinary_Robust* output values for *p.value_singlevariant*. Odds ratios were calculated for the number of variant carriers at the relevant rare variant or region using Fisher’s Exact test. Region-based tests for severe obesity (BMI > 40 kg/m^2^) as a binary trait were performed as for gene-based tests after restricting to the regions defined in Supplementary Table 7; burden and SKAT-O p-values should be interpreted with caution due to the small number of expected cases in the specified regions.

### UKBB 300 K exomes

This research been conducted using the https://genebass.org resource of summary statistics generated from the UK Biobank resource (under application 26041 and 48511). Briefly, genebass.org provides summary statistics for ~280,000 non-Finnish European (NFE) exomes using the SAIGE-GENE mixed model framework, including single-variant tests and gene-based burden (mean) and SKAT-O tests. We obtained summary statistics from https://genebass.org (accessed 6th Aug 2021) for BMI (UKB Field 21001), for anthropometry traits measured at UKB Assessment Centres, and for selected Early Life traits (comparison of height or body size at age 10).

### Open Targets Genetics GWAS/PheWAS loci and single-variant summary statistics

We used the Open Targets Genetics platform^22^ to obtain genome-wide significant GWAS/PheWAS loci for which a “locus-to-gene” (L2G) score was reported for GPR10 (https://genetics.opentargets.org/gene/ENSG00000119973, accessed 19th Aug 2021) and inspected whether GPR10 was prioritised as a putatively causal gene at loci associated with anthropometric traits or triglyceride levels (https://genetics.opentargets.org/study-locus/[studyIdentifier]/[variantIdentifier]).

We obtained single-variant summary statistics for GPR10 coding variant P305L (variant identifier 10-118594331-G-A, hg38) for anthropometric traits nominally associated with this variant (*P* < 0.05) in the UK Biobank (NEALE2/Neale v2, http://www.nealelab.is/uk-biobank/) or NHGRI-EBI GWAS Catalog^23^ (accessed 19th Aug 2021).

### cDNA constructs and site-directed mutagenesis

GPR10 WT cDNA was cloned into pCMV-Tag2B with an N-terminal FLAG tag and variant constructs were generated using QuickChange II XL site-directed mutagenesis kit (Agilent) according to the manufacturer’s recommendations. N-terminally cMyc-tagged GPR10 WT construct was generated by substitution of the FLAG tag in the above described pCMV-Tag2B construct using Q5® site-directed mutagenesis kit (New England Biolabs, Inc.) according to the supplier’s protocol. All constructs were verified by Sanger sequencing.

### Cell culture and transfection

COS7 cells were kindly provided by Professor Alan Tunnacliffe (Department of Chemical Engineering and Biotechnology, University of Cambridge, UK) and HEK293 cells were kindly provided by Professor Dario Alessi (MRC Protein Phosphorylation and Ubiquitylation Unit, University of Dundee, UK). HEK293 cells were authenticated via GENETICA Genotypes Analysis in May 2019, showing 97% match when compared to the reference profile ATCC sequence. COS-7 cells were not authenticated. Prior to the experiments, COS-7 and HEK293 cells were tested negative for mycoplasma contamination using MycoAlert enzymatic assay (LT07-703, Lonza) and MycoProbe Mycoplasma Detection kit (CUL001B, R&D Systems), respectively. COS-7 and HEK293 cells were maintained in low and high glucose Dulbecco’s modified eagle medium (Sigma-Aldrich, D6046; Gibco, 31966), respectively, supplemented with 10% fetal bovine serum (Gibco, 10270, South America origin), 1% GlutaMAX^TM^ (100X) (Gibco, 35050), and 100 units/mL penicillin and 100 μg/mL streptomycin (Sigma-Aldrich, P0781) and cultured at 37 °C in humidified air containing 5% CO_2_. Cells were transfected with respective cDNA constructs using Lipofectamine 2000™ (Gibco, 11668) in serum-free Opti-MEM medium (Gibco, 31985) according to the manufacturer’s protocols. For cell surface ELISA, competitive radioligand binding and inositol triphosphate turnover assays, COS-7 cells were seeded in poly-D-lysine (Sigma-Aldrich, P7886) coated 96-well plates at 20,000 cells/well density. Cells were transiently transfected using Lipofectamine 2000 (Invitrogen, 11668) according to manufacturer’s recommendations. In experiments using varying amounts of cDNA to assess the dominant negative effect of GPR10 mutants, the total amount of cDNA used in each transfection was maintained at 10 ng/well by topping up with empty vector.

### Generation of GPR10 stably expressing cell line

HEK293 cells stably expressing WT GPR10 were generated by calcium precipitation transfection with the cDNA construct described above that contains Neomycin resistance gene allowing selection in eukaryotic cells. Briefly, 20 µg GPR10 WT cDNA diluted in Tris-EDTA (TE) buffer supplemented with 250 uM CaCl_2_ was precipitated into an equal volume of 2x concentrated HEPES-buffered saline (HBS buffer) and added after 45 min dropwise onto cells scarcely seeded (i.e. approximately 30–50% confluence) in 10 cm diameter Petri dishes. After 24 h, cell culture medium was replaced with fresh media supplemented with 250 ug/ml Geneticin (G418, Sigma-Aldrich, G8168; effective dose was established in a killing curve experiment prior to generation of the stably expressing cells). Selection media was changed regularly every 1-2 days to assure the effective selection of the cells in which WT GPR10 and Neomycin resistance gene bearing plasmid were successfully integrated into the genome. These cells were then pooled and subsequently used for the cAMP accumulation assay described below.

### Cell surface expression ELISA

Cells transfected with N-terminal FLAG- or cMyc-tagged GPR10 constructs were tested for cell surface localization of the receptor with ELISA. A day after transfection, cells were fixed with 3.7% paraformaldehyde for 15 min at room temperature and washed three times with PBS. Subsequently, non-specific binding sites were blocked with 3% non-fat dry milk in 50 mM Tris-PBS pH 7.4 (blocking buffer) for 1 h at room temperature. Next, cells were incubated with a either a mouse monoclonal anti-FLAG antibody (Sigma-Aldrich, F1804) or anti-cMyc antibody (Millipore, CBL434, dilution 1:1000 in blocking buffer) overnight at 4 °C followed by triple washing with PBS and incubation with goat anti-mouse IgG(H + L)-HRP conjugate (Bio-Rad Laboratories, 172-1011) (1:1250 in 1.5% non-fat dry milk in 50 mM Tris-PBS pH 7.4) for 2 h at room temperature. Finally, plates were washed three times with PBS and a high performance chromogenic substrate, 3,3´,5,5´̵tetramethylbenzidine (TMB CORE+, Bio-Rad Laboratories, BUF062) was used to detect HRP activity. The reaction was terminated with 0.5 M H_2_SO_4_ and absorbance by the colour reaction product at 450 nm was determined using Tecan Infinite M1000 PRO microplate reader.

### Competition Radioligand binding assay

The effect of GPR10 mutants on PrRP-31 binding was assessed in a competition radioligand binding assay in intact, transiently transfected COS-7 cells. Cells were cultured in white 96-well plates and transiently transfected with 5 ng cDNA/well of GPR10 construct or empty vector (negative control) one day after seeding. Binding assays were performed approximately 24 h post transfection. For all following steps, cells were kept at 4 °C (on wet ice) and reagents were ice-cold to assure no receptor internalisation was induced by the agonist, causing undesired intracellular accumulation of radiolabeled tracer. Firstly, cells were washed (200 µl/well) and incubated with binding assay buffer (50 µl/well, 200 mM HEPES, pH 7.4 supplemented with 119 mM NaCl, 4.7 mM KCl, 5 mM MgCl_2_, 5.5 mM glucose, 1 mg/ml BSA). Varying doses of unlabelled PrRP-31 (1 pM – 1 µM) were added to the cells (5 µl/well), swiftly followed by dispensing 50 µl / well ^125^I-labelled PrRP-31 (Phoenix Pharmaceuticals Inc., T-008-50; 5 µl/ml dilution) and cells were incubated for 5 h at 4 °C. After washing twice with ice-cold binding assay buffer (200 µl/well), 25 µl/well 0.1 M NaOH was dispensed, cells were shaken for 2 min at 1000 rpm, followed by addition of 100 µl/well scintillation fluid, MicroScint-20 (Perkin Elmer, 6013621) and subsequent shaking for 5 min at 1000 rpm. Activity of ^125^I-PrRP-31 bound was quantified after at least 3 h settle time using a TopCount 9012 Microplate Liquid Scintillation Counter (Packard) through quantification of β-emission.

### cAMP accumulation assay

Measurement of ligand-induced cAMP generation in GPR10 WT stably expressing cells was done using HitHunter® cAMP assay (DiscoverX, 90-0075SM) according to manufacturer’s protocol with modifications. 20,000 cells/well were seeded in a white poly-D-lysine coated 96-well plate and cultured overnight. The following day, cells were washed with PBS and incubated in 30 μL PBS supplemented with 1 mM 3-­isobutyl-1-methylxanthine (IBMX, Cayman Chemical, 13347) for 30 min prior to stimulation with an agonist. Cells were stimulated with serial dilutions of PrRP-31 (1.5 μL/well, 20x dilution, Bachem, 4028740) for further 30 min at 37 °C. Intracellular cAMP detection was carried out directly after the ligand stimulation. 10 μL anti-cAMP antibody followed by 40 μL chemiluminescent substrate/lysis buffer/enzyme donor-cAMP complex mix prepared according to manufacturer’s protocol were added and plates were incubated shaking for 1 h at ambient temperature. Finally, 40 μL enzyme acceptor was dispensed and chemiluminescent signal was quantified after 4-5 h in a TopCount 9012 Microplate Counter (Packard).

### Inositol triphosphate turnover assay

COS-7 cells were transiently transfected with 5 ng/well GPR10 WT or variants cDNA construct, and in case of assays assessing Gα_i/_o-specific signalling only, co-transfected with additional 7.5 ng/well of Gα_Δ6qi4myr_ construct. Following the transfection, cells were cultured overnight in full media supplemented with 5 μl/ml [^3^H]-myo-inositol (Perkin Elmer, NET115600). After initial wash with Hank’s balanced salt solution (HBSS, Gibco, 14025), 100 μl/well of HBSS containing 10 mM LiCl (Sigma Aldrich, L9650) was added followed by stimulation with 5 μL/well 20x PrRP-31 stock solution for 75 min at 37 °C. Next, assay buffer was aspirated and cell were lysed with 50 μl/well 10 mM formic acid (Sigma Aldrich, F0507) while incubated on ice for at least 30 min. 20 μl lysate was transferred to a solid white 96-well plate containing 80 μl/well 12.5 mg/ml yttrium silicate poly-lysine-coated scintillation proximity beads (Perkin Elmer, RPNQ0010) suspension in water. Plates were sealed with a TopSeal-A PLUS (Perkin Elmer, 6050185), shaken vigorously at high speed for approximately 5 min and relative amount of radiolabeled IP1 was quantified after 8 h settle time using TopCount 9012 Microplate Counter (Packard).

### Analysis of in vitro data

All results from cell-based assays were analysed using GraphPad Prism 8 (GraphPad Software). Radioligand binding and signalling assays (i.e. cAMP accumulation and inositol triphosphate turnover assays) were performed in triplicates and were independently replicated at least three times. Results of each experiment were normalized to basal signal for mock transfected cells and maximal efficacy of PrRP-31 for GPR10 WT in a given assay as specified in a graph. Presented dose-response curves were plotted from the merged normalized data analysed with 3-parameter non-linear regression equation.

### Structure prediction and visualisation

Structural model of GPR10 WT was generated with a protein structure prediction service Robetta (http://www.robetta.org/) using Rosetta Comparative Modelling approach^24^. Generated structure was visualized and images were rendered using Open-Source PyMOL 1.8.x (https://www.lfd.uci.edu/~gohlke/).

### Generation of *Gpr10*^P193S^ mutant

We used CRISPR-Cas9 to generate the *Gpr10*^P193S^ mutation on a C57BL6/J background. *Gpr10* (*Prlhr*) is found on mouse chromosome 19. The sgRNA TGGTAGGTGTGCACCGCGGC-*CGG* targets the mutation site directly, and adheres to our stringent criteria for off target predictions (guides with mismatch (MM) of 0, 1 or 2 for elsewhere in the genome were discounted, and MM3 were tolerated if predicted off targets were not exonic) according to http://www.sanger.ac.uk/htgt/wge/). The sgRNA sequence was purchased as crRNA oligos, which were annealed with tracrRNA (both oligos supplied IDT; Coralville, USA) in sterile, RNase-free injection buffer (TrisHCl 1 mM, pH 7.5, EDTA 0.1 mM) by combining 2.5 µg crRNA with 5 µg tracrRNA and heating to 95 ^o^C, which was allowed to slowly cool to room temperature.

The following ssODN repair template, with capital letters indicating intended base pair changes was synthesised (IDT):

5’ctgaggctcagcgcctacgcggtgctgggcatctgggctctatctgcagtgctggcgctgTcggcTgcggtgcacacctaccatgtggagctcaagccccacgacgtgagcctctgcgag3’.

This repair template was designed to convert P193 CCG codon to a Serine TCG codon (Supplementary Fig. 4a). A second silent C > T mutation results in loss of an EagI restriction site for screening purposes.

CRISPR reagents (final concentrations; sgRNA 20 ng/µl, Cas9 protein 20 ng/µl, ssODN 50 ng/µl) were directly microinjected into C57BL6/J (Envigo, Bicester, UK) zygote pronuclei using standard protocols. Zygotes were cultured overnight and the resulting two-cell embryos surgically implanted into the oviduct of day 0.5 *post coitum* pseudopregnant mice. Potential founder mice were identified by extraction of genomic DNA from ear clips, followed by PCR using primers that flank the homology arms and sgRNA site (Geno F1 ttcacactcaccacaatcgc and Geno R1 tcactgacacccgtacgtaa). WT and HDR sequences both produce a 304 bp band, with the WT allele susceptible to EagI digest (note, NHEJ could also result in loss of EagI digest). Several candidate mice were identified (Supplementary Fig. 4b) and three were taken forward for sequencing. The product was amplified using high fidelity Phusion polymerase (NEB), gel extracted and subcloned into pCRblunt (Invitrogen, UK). Colonies were mini-prepped and Sanger sequenced with M13 Forward and Reverse primers, and aligned to predicted knock-in sequence (Supplementary Fig. 4c). Positive pups were bred with a WT C57BL6/J to confirm germline transmission and a colony established.

### Mouse phenotyping studies

All procedures were conducted in accordance with the United Kingdom Animals (Scientific Procedures) Act, 1986 (ASPA). All animal experiments were performed according to U.K. Home Office licensing laws and approved by the local Animal Welfare and Ethical Review Board (University of Manchester, UK). In all experiments mice were group housed unless otherwise stated in a 12:12 h light: dark cycle, at room temperature (22 ^o^C; 50–55% humidity). Mice were provided with *ad libitum* access to standard rodent chow unless otherwise stated (#801151 RM1-P; Special Diet Services, Essex, UK) and water.

The *Gpr10*^*-/-*^ null mouse (C57BL6/J background) was a kind gift from Dr Alain Stricker-Krongrad (Millennium Pharmaceuticals, Cambridge, USA). The Gpr10^P193S^ mutant was generated by the University of Manchester Genome Editing Unit, as described below.

For body growth curves *Gpr10*^*-/-*^ null mice (male *n* = 21; female *n* = 6) and *Gpr10*^+/+^ wild-type mice (male *n* = 25; female *n* = 6) were maintained on standard chow and weighed weekly. Homozygous *Gpr10*^P193S/P193S^ (male *n* = 7; female *n* = 5), heterozygous *Gpr10*^+/P193S^ (male *n* = 18; female *n* = 8) and wild-type *Gpr10*^+/+^ (male *n* = 9; female *n* = 8) mice were switched from standard chow to *ad libitum* access to 60% high energy diet (HED; #824054 RM 60% energy from fat; Special Diet Services, Essex, UK) at 8 weeks of age and weighed weekly for body growth curves.

In separate daily food intake studies, pre-obese *Gpr10*^-/-^ null and *Gpr10*^+/+^ wild-type mice (6 week old male *n* = 5 in each group; 8 week old female *n* = 6 in each group), and 8 week old homozygous *Gpr10*^P193S/P193S^ (male *n* = 11), heterozygous *Gpr10*^+/P193S^ (male *n* = 9) and wild-type *Gpr10*^+/+^ (male *n* = 9) were housed individually in standard cages with *ad libitum* access to standard chow. After at least a one week period of acclimatisation to single housing, daily food intake was measured over a minimum of 3 days at the same time each day and averaged to provide mean daily food take weight±SEM. Following initial food intake studies, *Gpr10*^-/-^ null and *Gpr10*^+/+^ wild-type mice (male *n* = 4 in each group; female *n* = 6 in each group) and homozygous *Gpr10*^P193S/P193S^ (male *n* = 11), heterozygous *Gpr10*^+/P193S^ (male *n* = 9) and wild-type *Gpr10*^+/+^ (male *n* = 9) were then acclimated to indirect calorimetric cages (Columbus Instruments, Columbus, OH, USA) for 2-3 days prior to study and data were collected for a minimum of 3 days during which time oxygen consumption (VO_2_ ml/hr) was measured every 8 min using Oxymax® software (Columbus Instruments, Columbus, OH, USA). Cages were not equipped with running wheels, and environmental enrichment was limited to bedding material. During this time all mice had *ad libitum* access to standard chow and water. Data were averaged every 30 min for continuous plots and day and night data averaged over the 3-day study period. Mice were then returned to their home cages and daily food intake and oxygen consumption were recorded for male and female *Gpr10*^-/-^ null and *Gpr10*^+/+^ wild-type mice during the same study at later stages; at 10, 14 and 18 weeks for male mice (*n* = 5 in each group), and at 25 weeks for female mice (*Gpr10*^+/+^ *n* = 6 and *Gpr10*^-/-^ null *n* = 5 respectively).

### Statistical analysis of in vivo data

Data are presented as means±SEM. Statistical analyses were performed using Prism statistical package (GraphPad Software Inc, San Diego, USA). Two-tailed Student’s *t*-tests or two-way ANOVA followed by Bonferroni’s multiple-comparison *post hoc* tests were used to compare two or three groups, respectively. Body-weight data were analysed with a mixed-effects analysis followed by Sidak’s multiple-comparison *post hoc* test.

### Reporting summary

Further information on research design is available in the Nature Portfolio Reporting Summary linked to this article.

## Supplementary information

Supplementary Information

Description of Additional Supplementary Files

Supplementary Data 1 -7

Reporting Summary

## Data Availability

All data are available in the main text, Supplementary Information, or source data file. Exome sequencing data are accessible from the European Genome-phenome Archive (https://ega-archive.org) under a managed access agreement for ethical issues (https://ega-archive.org/studies/EGAS00001000124). Data can only be used to interrogate obesity genes due to the nature of the consent obtained from participants. Please contact Sadaf Farooqi for any queries (isf20@cam.ac.uk); expected response time 1 month. Source data are provided with this paper.

## Source data

Source Data
