# Supplementary material for: A rare human variant that disrupts GPR10 signalling causes weight gain in mice
Source: Nat Commun. 2023 Mar 15;14:1450. doi: 10.1038/s41467-023-36966-3 (PMC10017677; doi:10.1038/s41467-023-36966-3)
Supplement: Supplementary file 1 — Supplementary Information [file 41467_2023_36966_MOESM1_ESM.pdf]

Supplementary Information

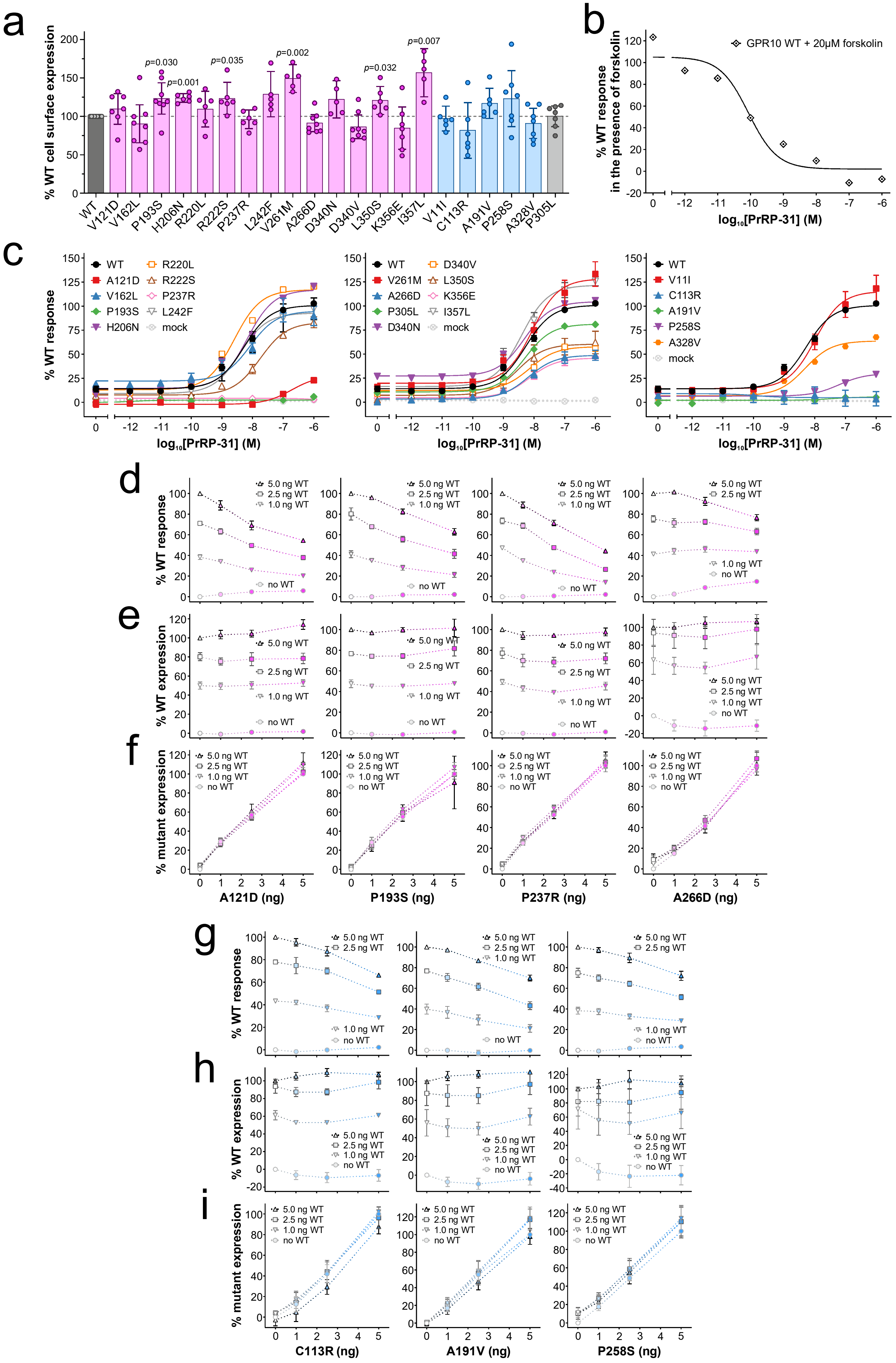
a
% WT cell surface expression
p=0.030
p=0.001
p=0.035
p=0.002
p=0.032
p=0.007
WT
V121D
V162L
P193S
H206N
R220L
R222S
P237R
L242F
V261M
A266D
D340N
D340V
L350S
K356E
I357L
V11I
C113R
A191V
P258S
A328V
P305L
b
GPR10 WT + 20μM forskolin
% WT response in the presence of forskolin
log10[PrRP-31] (M)
c
% WT response
WT
A121D
V162L
P193S
H206N
R220L
R222S
P237R
L242F
mock
V261M
A266D
P305L
D340N
D340V
L350S
K356E
I357L
V11I
C113R
A191V
P258S
A328V
log10[PrRP-31] (M)
d
% WT response
5.0 ng WT
2.5 ng WT
1.0 ng WT
no WT
e
% WT expression
f
% mutant expression
A121D (ng)
P193S (ng)
P237R (ng)
A266D (ng)
g
% WT response
h
% WT expression
i
% mutant expression
C113R (ng)
A191V (ng)
P258S (ng)

Supplementary Fig. 1 | Effect of GPR10 mutants on receptor expression and intracellular signalling. Functional studies of rare variants identified in individuals with severe early onset obesity (magenta) and in controls (blue). **a** Cell surface expression measured by ELISA; values expressed as % WT expression; mock transfected cells served as a negative control. Mean ± SEM of at least 5 experiments shown; statistical differences between means for WT and mutant GPR10 were compared with Students *t*-test, *p*-values lower than 0.05 provided. **b** Ligand (PrRP-31)-induced $G\alpha_{i/o}$ signalling by WT GPR10 in stably transfected HEK293 cells. Sum curves plotted from values normalized to maximal forskolin induced signal. Data from 3 (in the presence of forskolin) or 2 (without forskolin) independent experiments ± SEM. **c** Effects of GPR10 mutants on ligand (PrRP-31)-induced activation of $G\alpha_{i/o}$ pathway assessed using an inositol triphosphate turnover assay in COS-7 transiently co-transfected with $G\alpha_{\Delta 6qi4myr}$ chimeric G-protein. Data shown as sum curves from 3-7 experiments normalized to WT maximal response ± SEM. **d**-**i** Potential dominant negative effects of selected LoF GPR10 variants identified in obese cases (**d**-**f**) and controls (**g**-**i**) assessed in COS-7 cells transiently co–transfected with varying amounts of WT and mutant receptor. PrRP-31-induced $G\alpha_{q/11}$-coupled inositol triphosphate signalling (**d**, **g**) and cell surface expression of WT (**e**, **h**) vs mutant GPR10 (**f**, **i**) were measured; mean ± SEM of 3 experiments.

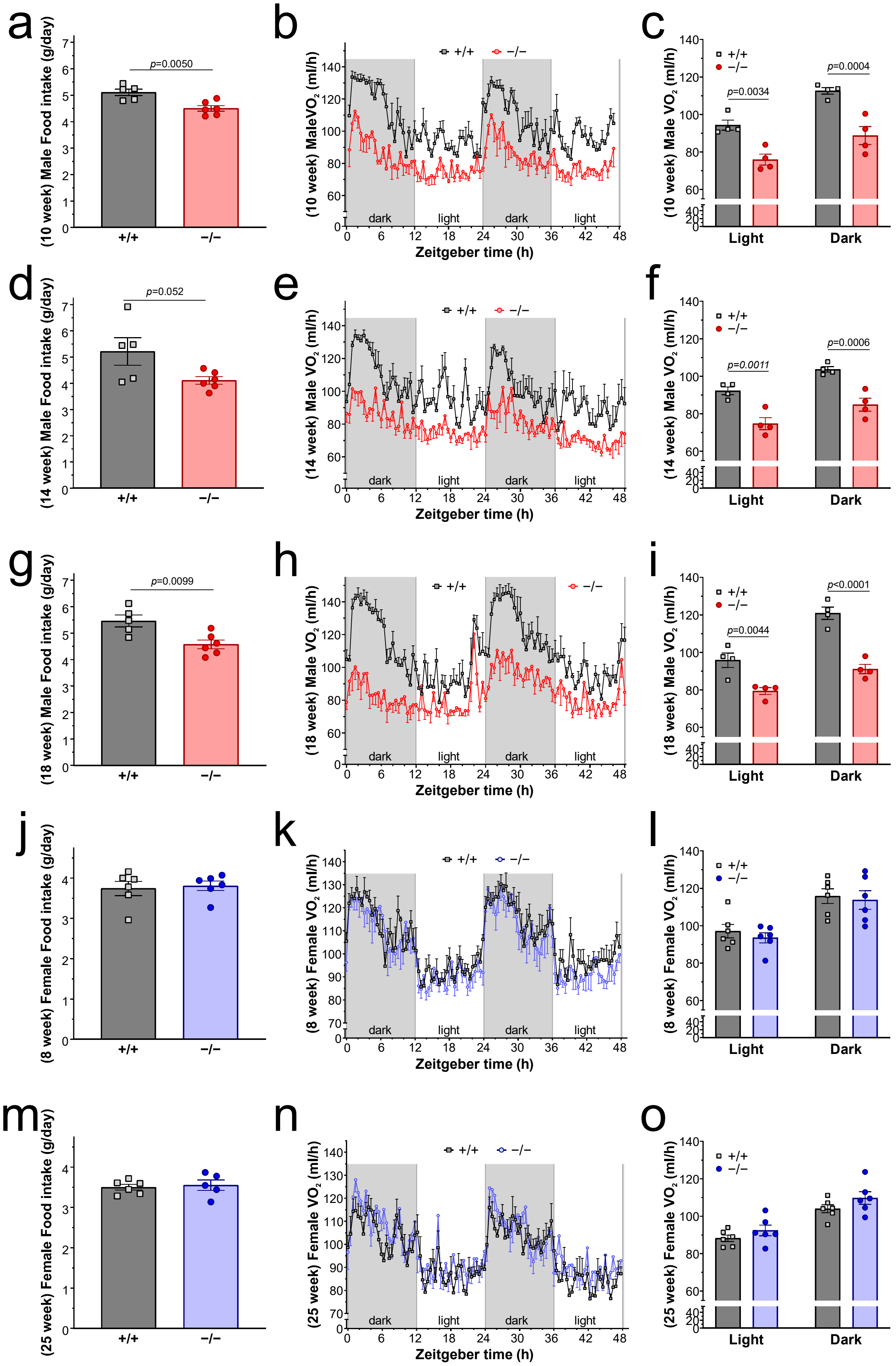

Supplementary Fig. 2 | Energy intake and expenditure in GPR10 knockout mice. Daily food intake (g) of male $Gpr10^{+/+}$ (grey squares) (n = 5) and $Gpr10^{-/-}$ null (red circles) (n = 6) mice at (**a**) 10 weeks, (**d**) 14 weeks and (**g**) 18 weeks. Oxygen consumption (VO_2, ml/hr) of male $Gpr10^{+/+}$ (grey squares) and $Gpr10^{-/-}$ null (red circles) mice (n = 4 in each group) at (**b**, **c**) 10 weeks, (**e**, **f**) 14 weeks and (**h**, **i**) 18 weeks**.** Daily food intake (g) for female $Gpr10^{+/+}$ (grey squares) and $Gpr10^{-/-}$ null (blue circles) mice aged 8 weeks (n = 6 in each group) (**j**) and 25 weeks (n = 5 and n = 6 respectively). (**m**) Oxygen consumption (VO_2, ml/hr) of female $Gpr10^{+/+}$ (grey squares) and $Gpr10^{-/-}$ null (blue circles) mice at (**k**, **l**) 8 weeks and (**n**, **o**) 25 weeks (n = 6 in each group). All data are presented as mean ± SEM and comparisons made using two-tailed Student's *t*-tests (**a**, **d**, **g**, **j**, **m**) or two-way ANOVA followed by Bonferroni's multiple-comparison *post hoc* tests (**c**, **f**, **i**, **l**, **o**) respectively.

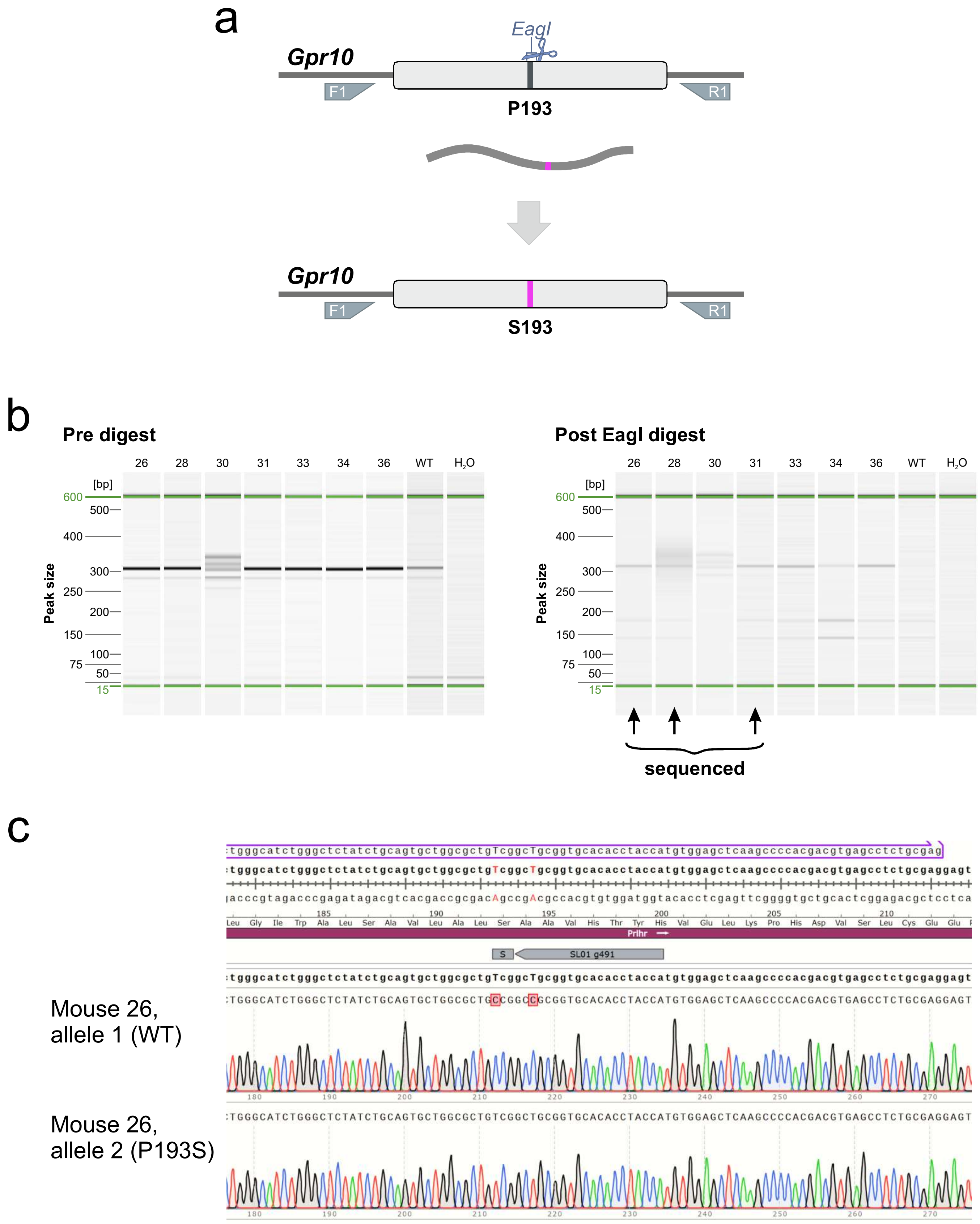

Supplementary Fig. 3 | Generation of transgenic mice carrying a human variant in *GPR10* (P193S)
a Design of P193S point mutation by CRISPR-Cas9. sgRNA targeting the P193 codon, indicated by scissors, combined with Cas9 protein and a ssODN donor template for embryo injection. **b** PCR genotyping, using primers flanking the target site and EagI digest identifying candidate pups. **c** Sanger sequencing and alignment against predicted knock-in sequence confirms knock-in for mouse 26 (a single founder shown for clarity).

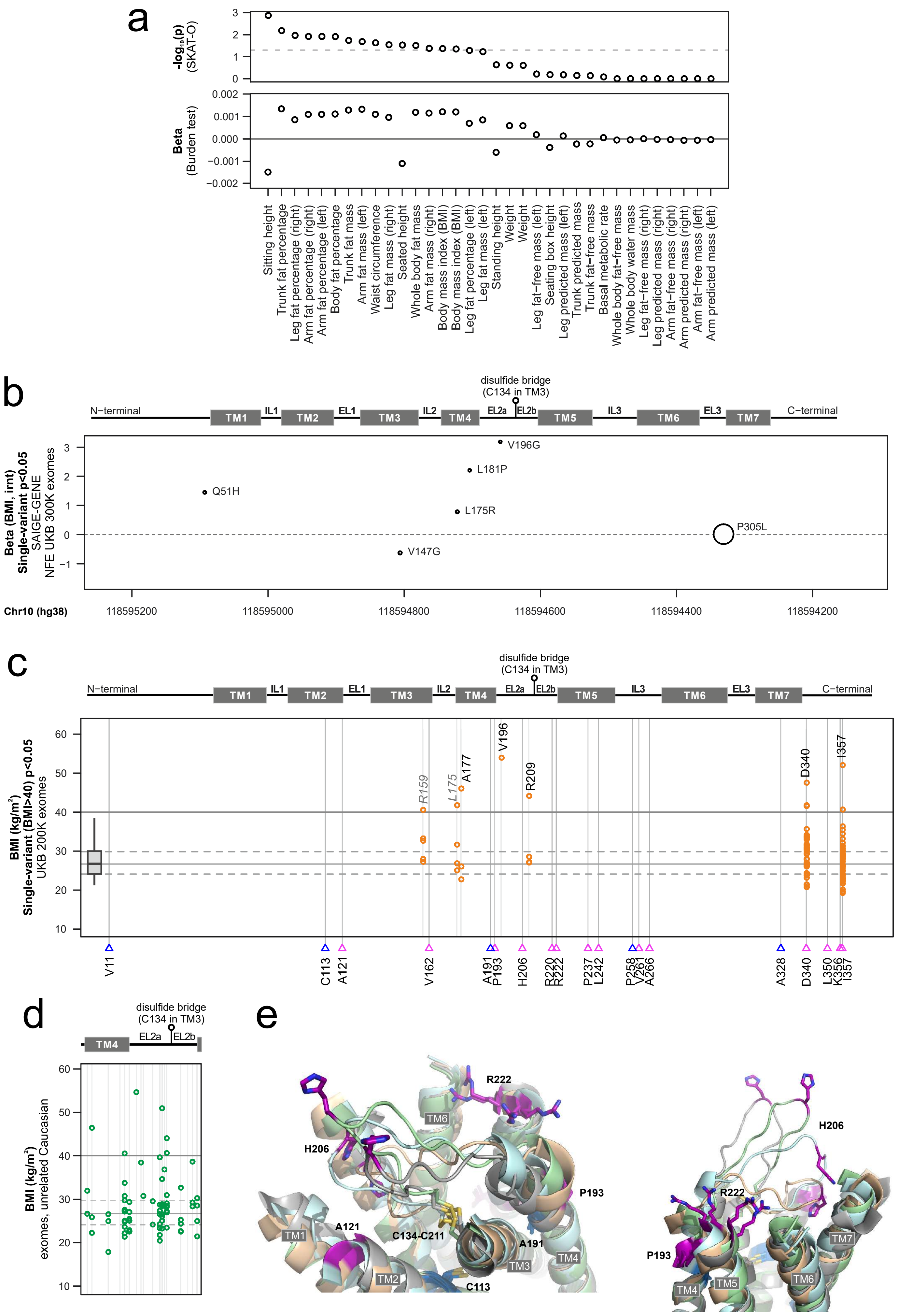
a
-log10(p) (SKAT-O)
Beta (Burden test)
Sitting height
Trunk fat percentage
Leg fat percentage (right)
Arm fat percentage (right)
Arm fat percentage (left)
Body fat percentage
Trunk fat mass
Arm fat mass (left)
Waist circumference
Leg fat mass (right)
Seated height
Whole body fat mass
Arm fat mass (right)
Body mass index (BMI)
Body mass index (BMI)
Leg fat percentage (left)
Leg fat mass (left)
Standing height
Weight
Weight
Leg fat-free mass (left)
Seating box height
Leg predicted mass (left)
Trunk predicted mass
Trunk fat-free mass
Basal metabolic rate
Whole body fat-free mass
Whole body water mass
Leg fat-free mass (right)
Leg predicted mass (right)
Arm fat-free mass (right)
Arm predicted mass (right)
Arm fat-free mass (left)
Arm predicted mass (left)
b
disulfide bridge (C134 in TM3)
N-terminal TM1 IL1 TM2 EL1 TM3 IL2 TM4 EL2a EL2b TM5 IL3 TM6 EL3 TM7 C-terminal
Beta (BMI, irnt) Single-variant p<0.05 SAIGE-GENE NFE UKB 300K exomes
V196G
L181P
Q51H
L175R
P305L
V147G
Chr10 (hg38)
118595200 118595000 118594800 118594600 118594400 118594200
c
disulfide bridge (C134 in TM3)
N-terminal TM1 IL1 TM2 EL1 TM3 IL2 TM4 EL2a EL2b TM5 IL3 TM6 EL3 TM7 C-terminal
BMI (kg/m²) Single-variant (BMI>40) p<0.05 UKB 200K exomes
R159 L175 A177 V196 R209 D340 I357
V11 C113 A121 V162 A191 P193 H206 R220 R222 P237 L242 P258 V261 A266 A328 D340 L350 K356 I357
d
disulfide bridge (C134 in TM3)
TM4 EL2a EL2b
BMI (kg/m²) exomes, unrelated Caucasian
e
R222
TM6
H206
P193
TM1
A121
C134-C211
A191
TM4
TM3
TM2
C113
H206
R222
P193
TM7
TM4
TM5
TM6

Supplementary Fig. 4 | *GPR10* coding variants in UK Biobank 200K and 300K exomes. a Gene-based summary statistics for anthropometric traits using *GPR10* missense variants in ~280K Non-Finnish Europeans from UKB 300K exomes. Summary statistics were obtained from https://genebass.org and are provided in Supplementary Tables S5-7. UK Biobank Field IDs for each point are provided in in Supplementary Table S5. **b** Effect size (beta) for variants with nominal $p<0.05$ from single-variant summary statistics for continuous BMI (kg/m^2) in ~280K Non-Finnish Europeans from UKB 300K exomes. Summary statistics were obtained from https://genebass.org. Point sizes are inversely proportional to allele frequency. Protein domains shown in schematic (top): TM, transmembrane domain; IL, intracellular loop, EL, extracellular loop; H8, helix 8. Horizontal grey lines show the median (solid), IQR (dashed) and whiskers (dotted) of BMIs among unrelated European exomes. **c** Scatterplot of body mass index (BMI) versus amino acid position in ~180K unrelated UK Biobank OQFE exomes, showing variants with single-variant nominal $p<0.05$ in gene-based Robust Burden test for rare nonsynonymous *GPR10* variants (MAF<0.1%) versus severe obesity (BMI>40kg/m^2). Five of the seven variants also had nominal $p<0.05$ in the European-only analysis (A177P, V196G, R209S, D340N, I357R). Boxplot illustrates median, upper and lower quartiles (box) and quartiles +/- 1.5 IQR (whiskers) in this cohort. Protein domain schematic as for (**b**). Triangles (bottom) indicate the position of variants functionally investigated in this study (from Figure 1a; magenta: cases, blue: controls; labelled as reference amino acid and position). **d** BMI (kg/m^2) of people harbouring a missense variant in *GPR10* transmembrane domain 4 (TM4), extracellular loops 2a (EL2a) or 2b (EL2b), among unrelated Europeans from UK Biobank 200K OQFE exomes. Region-based tests of severe obesity (BMI>40kg/m^2) as a binary trait for specified protein domains are provided in Supplementary Table S7. **e** Homology model of GPR10 generated with Robetta server (http://www.robetta.org/). Backbone of transmembrane (TM) domains, extracellular loop (EL) 2 section spanning TM4 with TM3 through a Cys134 – Cys211 disulphide bridge; selected amino acid side chains affected by genetic variants (blue) are shown in two different projections from the extracellular side.
