# Supplementary material for: A rare human variant that disrupts GPR10 signalling causes weight gain in mice
Source: Nat Commun. 2023 Mar 15;14:1450. doi: 10.1038/s41467-023-36966-3 (PMC10017677; doi:10.1038/s41467-023-36966-3)
Supplement: Supplementary file 2 — Description of Additional Supplementary Files [file 41467_2023_36966_MOESM2_ESM.pdf]

Description of Additional Supplementary Files

File Name: Supplementary Data 1
Description: GPR10 variants included in functional studies.

File Name: Supplementary Data 2
Description: Cell surface expression and signalling properties of GPR10 mutants.

File Name: Supplementary Data 3
Description: Open Targets Genetics "Locus2Gene" scores reported for GPR10 (PRLHR) at GWAS or PheWAS loci for anthropometric and related traits.

File Name: Supplementary Data 4
Description: Open Targets Genetics PheWAS browser results for GPR10 coding variant P305L.

File Name: Supplementary Data 5
Description: Single-variant association tests for GPR10 coding variant P305L in UK Biobank.

File Name: Supplementary Data 6
Description: Gene-based SKAT-O and burden tests for GPR10 missense variants in UK Biobank.

File Name: Supplementary Data 7
Description: Analysis of rare coding variants in GPR10 protein domains in UK Biobank exomes.
