# Supplementary material for: A rare human variant that disrupts GPR10 signalling causes weight gain in mice
Source: Nat Commun. 2023 Mar 15;14:1450. doi: 10.1038/s41467-023-36966-3 (PMC10017677; doi:10.1038/s41467-023-36966-3)
Supplement: Supplementary file 4 — Reporting Summary [file 41467_2023_36966_MOESM4_ESM.pdf]

nature portfolio

Corresponding author(s): Professor I. Sadaf Farooqi
Professor Simon M. Luckman

Last updated by author(s): Jan 24, 2023

Reporting Summary

Nature Portfolio wishes to improve the reproducibility of the work that we publish. This form provides structure for consistency and transparency in reporting. For further information on Nature Portfolio policies, see our Editorial Policies and the Editorial Policy Checklist.

Statistics

For all statistical analyses, confirm that the following items are present in the figure legend, table legend, main text, or Methods section.

n/a	Confirmed	
☐	☒	The exact sample size (*n*) for each experimental group/condition, given as a discrete number and unit of measurement
☐	☒	A statement on whether measurements were taken from distinct samples or whether the same sample was measured repeatedly
☐	☒	The statistical test(s) used AND whether they are one- or two-sided<br>*Only common tests should be described solely by name; describe more complex techniques in the Methods section.*
☐	☒	A description of all covariates tested
☐	☒	A description of any assumptions or corrections, such as tests of normality and adjustment for multiple comparisons
☐	☒	A full description of the statistical parameters including central tendency (e.g. means) or other basic estimates (e.g. regression coefficient) AND variation (e.g. standard deviation) or associated estimates of uncertainty (e.g. confidence intervals)
☐	☒	For null hypothesis testing, the test statistic (e.g. *F*, *t*, *r*) with confidence intervals, effect sizes, degrees of freedom and *P* value noted<br>*Give P values as exact values whenever suitable.*
☒	☐	For Bayesian analysis, information on the choice of priors and Markov chain Monte Carlo settings
☒	☐	For hierarchical and complex designs, identification of the appropriate level for tests and full reporting of outcomes
☐	☒	Estimates of effect sizes (e.g. Cohen's *d*, Pearson's *r*), indicating how they were calculated

Our web collection on statistics for biologists contains articles on many of the points above.

Software and code

Policy information about availability of computer code

Data collection	Comprehensive lab animal monitoring system (CLAMS) (Linton Instrumentation, Linton, UK, and Columbus Instruments, Columbus, OH), Oxymax software (Linton Instrumentation, Linton, UK, and Columbus Instruments, Columbus, OH), energy expenditure (O2 consumption).
Data analysis	All results from animal work and cell-based assays were analysed using GraphPad Prism version 8 (https://www.graphpad.com).<br><br>Gene-based burden and SKAT-O tests were performed using R v3.6.1 (https://www.r-project.org), package SKAT v2.0.1 (https://cran.r-project.org/src/contrib/Archive/SKAT/SKAT_2.0.1.tar.gz ; link to latest version is https://cran.r-project.org/web/packages/SKAT/index.html for latest versions)<br><br>Protein structure prediction and visualisation performed in Robetta (http://www.robetta.org/) and Open-Source PyMOL 1.8.x (https://www.lfd.uci.edu/~gohlke/)

For manuscripts utilizing custom algorithms or software that are central to the research but not yet described in published literature, software must be made available to editors and reviewers. We strongly encourage code deposition in a community repository (e.g. GitHub). See the Nature Portfolio guidelines for submitting code & software for further information.

Data

Policy information about availability of data

All manuscripts must include a data availability statement. This statement should provide the following information, where applicable:

- Accession codes, unique identifiers, or web links for publicly available datasets
- A description of any restrictions on data availability
- For clinical datasets or third party data, please ensure that the statement adheres to our policy

All data are available in the main text, Supplementary Information, or source data file. Exome sequencing data are accessible from the European Genome-phenome Archive (https://ega-archive.org) under a managed access agreement (EGAS00001000124). Source data are provided with this paper.

Field-specific reporting

Please select the one below that is the best fit for your research. If you are not sure, read the appropriate sections before making your selection.

☒ Life sciences ☐ Behavioural & social sciences ☐ Ecological, evolutionary & environmental sciences

For a reference copy of the document with all sections, see nature.com/documents/nr-reporting-summary-flat.pdf

Life sciences study design

All studies must disclose on these points even when the disclosure is negative.

Sample size	In this study, we sought to determine the clinical phenotype associated with rare genetic mutations in GPR10. For this reason, all participants harbouring rare variants were included. In animal studies sample size was determined based on similar studies in this field and using power analysis.
Data exclusions	No data was excluded.
Replication	Rare variants identified by exome sequencing were validated by Sanger sequencing. All mutations were validated using this method.<br>For in vitro experiments, number of replicates has been clearly stated in figure legends and/or supplementary tables.
Randomization	This was an observational and mechanistic study, therefore randomization was not required.
Blinding	For human data collection, investigators were not blinded. Blinding during collection was not needed because conditions were well controlled. Blinding is also not necessary because the results are quantitative and did not require subjective judgment or interpretation.<br><br>For animal studies, where possible the experimenter who conducted the experiments was blind to the genotype and grouping of the animals. Again results are quantitative and did not require subjective judgment or interpretation.

Reporting for specific materials, systems and methods

We require information from authors about some types of materials, experimental systems and methods used in many studies. Here, indicate whether each material, system or method listed is relevant to your study. If you are not sure if a list item applies to your research, read the appropriate section before selecting a response.

Materials & experimental systems

n/a	Involved in the study	
☐	☒	Antibodies
☐	☒	Eukaryotic cell lines
☒	☐	Palaeontology and archaeology
☐	☒	Animals and other organisms
☐	☒	Human research participants
☒	☐	Clinical data
☒	☐	Dual use research of concern

Methods

n/a	Involved in the study	
☒	☐	ChIP-seq
☒	☐	Flow cytometry
☒	☐	MRI-based neuroimaging

Antibodies

Antibodies used	Supplier name, Catalog number, clone name, lot number.<br>Mouse anti-FLAG M2 antibody (dilution 1:1000) (F1804, Sigma-Aldrich)<br>Mouse anti-cMyc antibody clone 3C7 (dilution 1:1000) (CBL434, Millipore)<br>Goat anti-mouse IgG(H+L)-HRP conjugate (dilution 1:1254880) (172-1011, Bio-Rad Laboratories)

Anti-β-endorphin (#H-022-33, Phoenix Peptide)
Anti-c-Fos (Ab208942, Abcam)
AlexaFluor 488 -conjugaeted secondary antibody (A21206, Invitrogen)
AlexaFluor 594 -conjugated secondary antibody (A21203, Invitrogen)

Validation

The anti-β-endorphin (#H-022-33, Phoenix Peptide) has been validated in several previous work (listed in the website) and validated in our previous work (He et al., 2021). Anti-c-Fos (Ab208942, Abcam) antibody has been validated by Abcam (https://www.abcam.com/c-fos-antibody-2h2-ab208942.html) and also validated in our previous work (He et al., 2021).
Anti-FLAG and anti-cMyc are standard commercially available antibodies and validated by Sigma-Aldrich (https://www.sigmaaldrich.com/GB/en/product/sigma/f1804) and Millipore (https://www.merckmillipore.com/GB/en/product/Anti-c-myc-Antibody-clone-3C7,MM_NF-CBL434), respectively.

Eukaryotic cell lines

Policy information about cell lines

Cell line source(s)

HEK293 cells were kindly provided by Professor Dario Alessi (MRC Protein Phosphorylation and Ubiquitylation Unit, University of Dundee) and COS7 cells were kindly provided by Professor Alan Tunnacliffe (Department of Chemical Engineering and Biotechnology, University of Cambridge)

Authentication

HEK293 cells were authenticated via GENETICA Genotypes Analysis in May 2019, showing 97% match when compared to the reference profile ATCC sequence.
COS7 cells were not authenticated.

Mycoplasma contamination

HEK293 cells were tested negative for mycoplasma contamination using MycoProbe Mycoplasma Detection Kit (CUL001B, R&D Systems) and COS7 cells were tested negative for mycoplasma contamination using MycoAlert enzymatic assay (LT07-703, Lonza).

Commonly misidentified lines
(See ICLAC register)

None

Animals and other organisms

Policy information about studies involving animals; ARRIVE guidelines recommended for reporting animal research

Laboratory animals

We used laboratory mice as the model system. All study mice are bred on a C57Bl6J background.

GPR10-/- null and GPR10+/+ wild-type group housed male (n = 21 and n = 25 respectively) and female (n=6 in each group) mice were used for long term body weight studies.
Daily food intake of standard chow was measured in singly housed pre obese 6 week old GPR10-/- null and GPR10+/+ wild-type male mice (n=6 and n=5 respectively), and again at 10, 14 and 18 weeks old (n=5 and n=6 respectively).
Oxygen consumption was measured in pre obese 6 week old GPR10-/- null and GPR10+/+ wild-type mice male mice (n=5 in each group), and again at 10, 14 and 18 weeks old (n=4 in each group).
Daily food intake of standard chow was measured in pre obese 8 week old GPR10-/- null and GPR10+/+ wild-type female mice (n=6 in each group), and again at 25 weeks old (n=5 and n=6 respectively).
Oxygen consumption was measured in pre obese 8 week old, and again at 25 weeks old GPR10-/- null and GPR10+/+ wild-type mice female mice (n=6 in each group).

Homozygous GPR10P193S/P193S (male n = 7; female n = 5), heterozygous GPR10+/P193S (male n = 18; female n = 8) and wild-type GPR10+/+ (male n = 9; female n = 8) mice were switched from standard chow to ad libitum access to 60% high energy diet at 8 weeks of age and weighed weekly for body growth curves.
Daily food intake of standard chow was measured in 8 week old homozygous GPR10P193S/P193S (male n = 11), heterozygous GPR10+/P193S (male n = 9) and wild-type GPR10+/+ (male n = 9).
Oxygen consumption was measured in homozygous GPR10P193S/P193S (male n = 11), heterozygous GPR10+/P193S (male n = 9) and wild-type GPR10+/+ (male n = 9).

Wild animals

No wild animals were used in this study.

Field-collected samples

No field collected samples were used in this study

Ethics oversight

All procedures were conducted in accordance with the United Kingdom Animals (Scientific Procedures) Act, 1986 (ASPA) All animal experiments were performed according to U.K. Home Office licensing laws and approved by the local Animal Welfare and Ethical Review Board (University of Manchester, UK).

Note that full information on the approval of the study protocol must also be provided in the manuscript.

Human research participants

Policy information about studies involving human research participants

Population characteristics

Male and female participants (sex noted by Physicians) were recruited from the Genetics of Obesity Study (www.goos.org.uk) were referred by their physicians based on the following criteria:
(1) Early onset obesity (before age 10)

	(2) Severe obesity as defined by BMI ≥ 3 standard deviation scores.<br>All participants found to have missense mutations in HTR2C and their family members were invited to participate.
Recruitment	Participants were referred to the Genetics of Obesity Studies by their physicians if they satisfied the criteria listed above, irrespective of their physical location.
Ethics oversight	All studies were approved by the Multi-Regional Ethics Committee and the Cambridge Local Research Ethics Committee (MREC 97/21 and REC number 03/103). All participants, or their legal guardian for those aged under 16, provided written consent for all assessments; participants under the age of 16 provided oral assent. Subjects cannot be identified through the information included in this paper.

Note that full information on the approval of the study protocol must also be provided in the manuscript.
